# Cold Exposure Exacerbates the Development of Diabetic Polyneuropathy in the Rat

**DOI:** 10.1155/2009/827943

**Published:** 2010-01-14

**Authors:** Lora J. Kasselman, Aristidis Veves, Christopher H. Gibbons, Seward B. Rutkove

**Affiliations:** ^1^Division of Neuromuscular Diseases, Department of Neurology, Beth Israel Deaconess Medical Center, 330 Brookline Avenue, Boston, MA 02215, USA; ^2^Department of Neurology, Harvard Medical School, Boston, MA 02115, USA; ^3^Department of Vascular Surgery, Beth Israel Deaconess Medical Center, 330 Brookline Avenue, Boston, MA 02215, USA

## Abstract

Diabetic polyneuropathy (DPN) and cold-induced nerve injury share several pathogenic mechanisms. This study explores whether cold exposure contributes to the development of DPN. Streptozotocin-induced diabetic rats and controls were exposed to a room temperature (23°C) or cold environment (10°C). H-reflex, tail and sciatic motor, and sensory nerve conduction studies were performed. Analyses of sural nerve, intraepidermal nerve fibers, and skin and nerve nitrotyrosine ELISAs were performed. Diabetic animals exposed to a cold environment had an increased H-reflex four weeks earlier than diabetic room temperature animals (*P* = .03). Cold-exposed diabetic animals also had greater reduction in motor conduction velocities at 20 weeks (*P* = .017), decreased skin nerve fiber density (*P* = .037), and increased skin nitrotyrosine levels (*P* = .047). Cold exposure appears to hasten the development of DPN in the rat STZ model of diabetes. These findings support that further study into the relationship between ambient temperature and DPN is warranted.

## 1. Introduction

In humans, brief periods of intense cold exposure can be tolerated without adverse consequences, though prolonged or repeated cold exposure can produce permanent nerve injury [1]. First described in ancient times, cold-induced pathology continues to be common, especially during times of war [2]. In addition, cold exposure can occur during normal daily activities such as being outdoors during the winter months. Even short exposures to mildly cold temperatures can reduce skin temperature in the periphery [3]. The mechanisms of cold-induced nerve injury are mainly ischemic, and include slugging of blood due to decreased velocity [4] and, with repeated exposure, reactive oxygen species formation [5]. However, more mild cold exposure can also impair other normal processes such as axonal transport [6, 7] and exacerbate potentially detrimental processes such as protein kinase C activation [8].

Many of these same mechanisms are also considered to be important in the pathogenesis of diabetic polyneuropathy (DPN) [9]. In particular, one area of overlap between cold exposure and diabetes is the increased susceptibility of diabetic nerves to ischemic damage [10, 11]. Because of these shared potential mechanisms, it is plausible that exposure to cold could enhance or perhaps accelerate the development of DPN, and that intentional warming of the distal extremities could protect against its development [12]. The implications of such a connection would be important since it could lead to new nonpharmacologic approaches toward slowing the development of DPN, substantially impacting diabetic patient care.

Whereas human epidemiological studies could help address this question, animal models may also provide a straightforward means for assessing the power of such an association. Here we report the results of a study evaluating the effects of repeated periods of cold exposure on the development of DPN in the streptozotocin-(STZ-)induced diabetic rat.

## 2. Materials and Methods

### 2.1. Animals and Induction of Diabetes

A total of 26 male Wistar rats (300–350 g; Charles River Laboratories, Wilmington, MA, USA) were acclimated for one week prior to experiments on a 12:12 hour light/dark cycle (lights on 07:00) and allowed food and water *ad libitum*. All of the following procedures were approved by Beth Israel Deaconess Medical Center's Institutional Animal Care and Use Committee and followed the National Institutes of Health's (NIH) “Principles of laboratory and animal care” guidelines.

Streptozotocin (STZ; Fisher Scientific, Houston, TX, USA) was injected into the tail vein of 12 rats, 10–12 weeks old, at a dose of 55 mg/kg body weight. The other 14 age-matched rats received an equal volume of sterile phosphate buffered saline. Forty-eight hours after injection of STZ, blood glucose levels were tested to confirm diabetes using a commercially available blood glucose kit (Fisher Scientific, Houston, TX, USA), and were monitored once weekly during the experiment. In addition to blood glucose, weight was measured weekly and hemoglobin A1c (HbA_1c_) values were determined twice during the study period. All animals were confirmed diabetic (plasma glucose levels of >250 mg/dL) 1 week after STZ injection. Following confirmation of diabetes, both diabetic and nondiabetic groups were each divided into two groups: one to be exposed to cold and the other to be exposed to room temperature. Exposure to these two environments began within one week of diabetes being successfully induced. Thus, this study had the following 4 groups: nondiabetic room temperature (*n* = 7), nondiabetic cold (*n* = 7), diabetic room temperature (*n* = 6), and diabetic cold (*n* = 6).

### 2.2. Cold Exposure

Chambers adapted from studies of cold nociception were used for all temperature exposures [13]. A clear plexiglass perimeter wall (15 cm high) was affixed to an aluminum plate (46 × 31 × 3 cm) and divided into six chambers by opaque black plexiglass dividers (15 × 15 cm  per chamber) each of which would hold a single animal. For cold exposure, the aluminum plate was cooled to 10 ± 2°C for 3 hours over an ice bath. This cold temperature range was chosen because preliminary data have suggested that normal human foot temperature can drop into this range during outdoor winter activities (Rutkove, unpublished results). More importantly, this represents a temperature far above that considered likely to produce permanent cold-injury (usually below 4°C) [4]. 

The room temperature chamber was identical to the one described above, but maintained at 23 ± 2°C for 3 hours using a room temperature water bath. Both chambers were constantly monitored with a differential thermocouple thermometer (Product #15-077-26, Fisher Scientific, Pittsburgh, PA, USA) embedded in the aluminum plates. Using this approach, ambient air temperature in the chambers remained between 22 and 23°C but occasionally decreased to 20°C during cold exposure. Animals were allowed to move freely within their chambers and were exposed to their respective conditions for approximately 3 hours/day, for 5 days/week for 20 weeks. The 3-hour duration of cold exposure was based upon the previous work assessing the impact of nonfreezing cold on the rat sciatic nerve [4]. The 5 days per week frequency was chosen as a matter of convenience and was reduced to 3-4 days if other tests (e.g., electrophysiology) were being performed on the animals in the same week.

### 2.3. Animal Temperature Measurements

Core, paw, and tail temperatures were measured before, during, and after exposure periods using a calibrated thermocouple (Product #15-077-26, Fisher Scientific, Pittsburgh, PA, USA). Paw temperature measurements were taken by placing the thermocouple probe between the 4th and 5th digits of the left hind paw. Core temperatures were measured rectally. Tail temperatures were measured by placing the probe on the proximal tail.

### 2.4. Electrophysiology

All electrophysiological measurements were taken from both hind limbs on days when the rats were not exposed to cold, with the rat limb temperature carefully maintained at a minimum of 34°C during testing using thermostatically controlled heating blankets. Electrophysiological measurements were recorded using a TECA Synergy T2 EMG Monitoring System and TECA accessories (Viasys, Madison, WI, USA); a common reference (ground) electrode was placed on the contralateral limb. For all studies, supramaximal stimuli were applied and velocities calculated from the onset latency of the recorded potential. All sensory studies were digitally averaged until a clear waveform was obtained.

#### 2.4.1. Sensory and Motor Nerve Conduction Tests in the Tail

For motor nerve conduction tests, a stimulating (cathode) needle electrode was placed in two positions, the second position 5 cm proximal to the first position. The recording electrode was placed distally. Orthodromic sensory studies were performed by employing the motor recording electrode described above as the stimulating electrode and recording with the more proximally placed motor stimulating electrodes.

#### 2.4.2. Sensory and Motor Nerve Conduction Tests in the Hind Limb (Sciatic-Tibial Nerves)

For motor nerve conduction tests, a cathodal needle electrode was placed in two positions: the first position was at the sciatic notch and the second position 3 cm distally. The recording needle electrode was placed in the foot. Orthodromic sensory studies were performed by using the motor recording electrode described above as the stimulating electrode and recording at the sciatic notch.

#### 2.4.3. H-Reflex Recording from the Hind Paw

These studies were performed on the sciatic-tibial nerve as previously described [14]. Briefly, a monopolar needle electrode was placed subcutaneously on the medial aspect of the ankle and was used as the stimulating electrode. An anodal needle electrode was placed proximally to the stimulating electrode on the lateral aspect of the leg. Another monopolar needle electrode was placed subcutaneously on the dorsomedial aspect of the hindpaw and used as the active recording electrode. A reference needle electrode was placed subcutaneously in the proximal region of the tail. Stimulus intensity was gradually increased until the H-reflex was at maximum amplitude. The latency of H-reflexes, if present, was recorded. Since the H-reflex is spinally mediated, the depth of anesthesia was carefully monitored, and reduced if necessary to assure the most robust response.

Electrophysiological measurements were conducted before the induction of diabetes to determine baseline values and repeated every other week for 20 weeks for tail motor and sensory nerves as well as H-reflex. Sciatic-tibial nerve conduction velocity was only conducted at one time point in order to compare values obtained in this study with other work in the literature.

### 2.5. Tissue Collection and Staining

Twenty weeks after beginning cold and room temperature exposure, all animals were given an overdose of chloral hydrate-pentobarbital and exsanguinated with heparinized saline at 4°C. Three millimeter skin punch biopsies were taken from the right hindpaws, lateral to the location of stimulating needle electrode placement; a sural nerve biopsy was taken at the calf level (the sural nerve was chosen over the sciatic-tibial nerve given its more superficial location and therefore higher likelihood of showing cold-induced injury). Both skin and nerve were postfixed in 2% paraformaldehyde-lysine-periodate in Sorrenson's buffer at 4°C for histology. Additional skin punch and sural nerve biopsies were taken from the left hind paw and calf, respectively, and flash frozen for ELISAs. Animals were then perfused with 2% glutaraldehyde, sural nerve tissue removed and post-fixed overnight at 4°C. Skin biopsies were cut on a sliding microtome at 50 *μ*m, and stored at −20°C. Nerve sections were cut on an ultramicrotome at 1 *μ*m and stained with Toluidine Blue. 

Skin sections were stained for PGP 9.5 using standard immunostaining techniques [15]. Briefly, sections were first incubated with rabbit-anti-PGP 9.5 (AbD Serotec, Raleigh, NC, USA) at 1: 10,000 overnight, followed by a biotinylated secondary antibody. The Vectastain Elite ABC immunoperoxidase kit (Vector Laboratories, Burlingame, CA, USA) and diaminobenzidene were used for visualization and sections were mounted using standard techniques.

### 2.6. Histological Quantification

*Skin. *PGP 9.5-stained skin sections were used to quantify intraepidermal nerve fiber (IENF) density. Observers blind to treatment group identified three areas of epidermis in three separate sections per animal, resulting in nine total areas used for IENF quantification using a previously described approach [15]. Images were captured and epidermal length was analyzed with the public domain NIH image program (http://rsb.info.nih.gov/nih-image/). IENF density was calculated by dividing the total number of fibers counted per section by the epidermal length of that section. 

*Sural Nerve. *Toluidine Blue-stained nerve sections were used to quantify axon density as well as the g-quotient, a ratio of axon thickness to total fiber thickness [16]; observers were blind to treatment group. A stereology grid (50 × 50 *μ*m) was overlaid on top of images captured at 40X; only axons and fibers that fell under the line intersections were counted and measured. Total nerve area, axon and fiber diameters were calculated using the NIH image program mentioned above. Briefly, NIH image was calibrated using an image of a stage micrometer with 10 *μ*m divisions taken at 40X. After line calibration, a straight line was used to measure axon diameter, from the inside edges of the myelin sheath, and fiber diameter, from the outside edges of the myelin sheath. Additionally, the total number of axons that fell under the line intersections were counted and the total nerve area was calculated by drawing a free-form line around the entire observable area seen at 40X. Axon density was quantified by dividing the total number of axons counted by total nerve area.

### 2.7. Nitrotyrosine ELISA

Flash frozen skin and nerve tissue were homogenized separately in lysis buffer. Total protein content was determined for each sample using a bicinchoninic acid (BCA) protein assay kit (ThermoScientific, Rockford, IL, USA). Homogenized tissue, normalized by total protein, was added to a nitrotyrosine coated 96-well plate from a nitrotyrosine ELISA kit (Cell Bioloabs, Inc., San Diego, CA, USA), in duplicate for both skin and nerve, and compared to nitrated bovine serum albumin standards. Anti-nitrotyrosine primary and horseradish peroxidase conjugated secondary antibodies from the ELISA kit were used at 1 : 1000. Results were read at 450 nm and optical density (OD) was used for statistical analysis.

### 2.8. Analysis

We analyzed all quantitative measures using SPSS version 16.0 for Windows (SPSS, Chicago, IL, USA). ANOVAs were conducted at a *P* ≤ .05 significance level using time as a repeated measure for weight, blood glucose levels, foot temperature, and electrophysiological measurements. For electrophysiological tests, no difference was detected between the left and right hind limb measurements so all analyses were conducted on data from the left side. Posthoc tests were conducted when appropriate. Any analyses done at a single time point (e.g., 20-week data and ELISA measurements) were completed using an ANOVA without time as a repeated measure. For histological analysis of skin and nerve, a hierarchical linear model was conducted at a *P* ≤ .05 significance level using random terms for animals and sections and a fixed term for treatment [17].

## 3. Results

### 3.1. Weight, Glucose, and  HbA_1c_

Baseline measurements of body weight, blood glucose, and HbA_1c_ were similar among all animals before random assignment to treatment groups. However, after induction of diabetes by STZ injection, diabetic animals did not gain weight, and remained significantly lighter than nondiabetic controls. In addition, diabetic animals had a significant increase in blood glucose and  HbA_1c_. There was no effect of cold exposure on body weight, blood glucose, or HbA_1c_ (Table 1).

### 3.2. Electrophysiology

**Figure 1:**
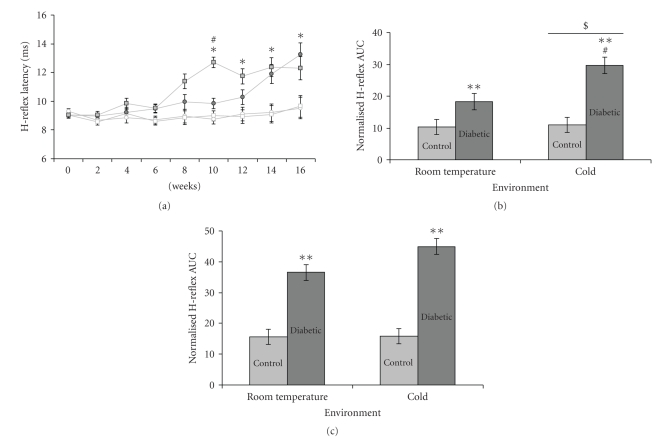
H-reflex latencies and area under the curve. (a) Graph of H-reflex latencies shows differences among the treatment groups (open circles = nondiabetic room temperature (*n* = 7), open squares = nondiabetic cold (*n* = 7), filled circles = diabetic room temperature (*n* = 6), and filled squares = diabetic cold (*n* = 6)). At 10 weeks, diabetic animals exposed to a cold environment had a significant increase in H-reflex latency as compared to diabetic animals exposed to a room temperature environment, nondiabetic animals exposed to a cold environment, and nondiabetic animals exposed to a room temperature environment. Mean ± SEM. ^#^ *P* = .03 versus all other groups. *P < .05 versus weeks 0–6. (b) Calculated area under the curve (AUC) of H-reflex latency from 0 to 10 weeks, derived from Figure 1(a). Diabetic animals had larger H-reflex latency AUCs than nondiabetic controls. Mean ± SEM. **P < .001 for diabetes, *$ P* = .025 for temperature, and ^#^ *P* = .042 for the interaction term. (c) Calculated AUC of H-reflex latency from 0 to 16 weeks derived from Figure 1(a). Although diabetic animals in both groups (room temperature and cold) had greater AUCs than nondiabetic controls, the temperature and interaction effects were no longer apparent. Mean ± SEM. **P < .001 versus nondiabetic controls.

H-reflex latency was similar among all animals at baseline. After 16 weeks, H-reflexes were typically absent in diabetic animals and therefore only data up to and including that time point were used for analysis. Overall, diabetic animals had a significantly longer H-reflex latency than nondiabetic animals (*P* = .001; see Figure 1(a)). In addition, after 10 weeks, diabetic animals exposed to a cold environment had a significantly longer H-reflex latency as compared to diabetic animals exposed to a room temperature environment and nondiabetic control animals (*P* = .03;  see Figure 1(a)), though while not significant, an increased latency in the diabetic cold animals could be seen as early as 8 weeks. In addition, at 10 weeks after STZ treatment, diabetic animals exposed to a cold environment had the largest H-reflex latency area under the curve (AUC) from 0 to 10 weeks (*P* = .042; see Figure 1(b)). However, by 16 weeks, diabetic animals exposed to a room temperature environment had a similar H-reflex AUC as diabetic animals exposed to a cold environment (*P* = .86;  see Figure 1(c)), though both groups of diabetic animals had larger AUCs than both nondiabetic control groups (*P* < .001;  see Figure 1(c)). There were no significant changes in the diabetic cold group compared to all other groups in any other H-reflex parameters, for example, amplitude or area (data not shown).

**Figure 2:**
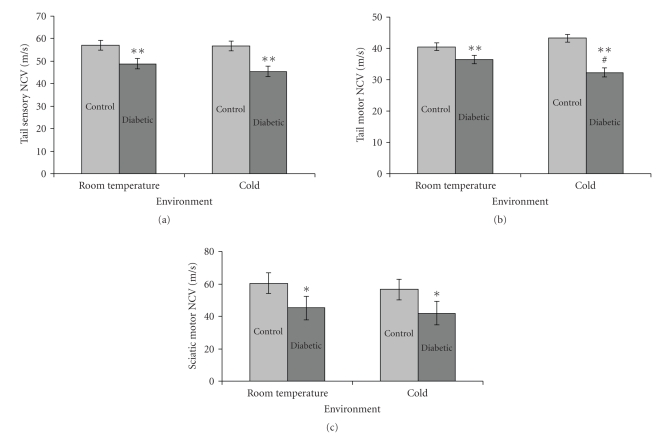
Tail and sciatic-tibial nerve conduction velocities. Graphs of sensory and motor nerve conduction velocities at 20 weeks show differences among the treatment groups (control room temperature (*n* = 7), control cold (*n* = 7), diabetic room temperature (*n* = 6), diabetic cold (*n* = 6)). (a) Diabetic animals (both room temperature and cold) had a significant decrease in tail sensory nerve conduction velocity as compared to nondiabetic control animals in both the room temperature and cold exposed groups. (b) Diabetic animals (both room temperature and cold) had a significant decrease in tail motor nerve conduction velocity as compared to nondiabetic control animals in both the room temperature and cold exposed groups. Additionally, diabetic animals exposed to a cold environment had a modest but significant reduction in conduction velocity compared the diabetic animals not exposed to cold. (c) Diabetic animals (both room temperature and cold) had a significant decrease in sciatic-tibial motor nerve conduction velocity compared to nondiabetic control animals in both the room temperature and cold exposed groups. Mean ± SEM. **P < .001 versus nondiabetic control animals, *P = .007 versus nondiabetic control animals, ^#^ *P* = .017 versus control room temperature and control cold exposed animals.

There were no differences in tail sensory or motor nerve conduction velocities at baseline. Similar to H-reflex latency, after treatment, both groups of diabetic animals had significant slowing of tail sensory nerve conduction velocity (week 20 data shown; *P* < .001; see Figure 2(a)) and tail motor nerve conduction velocity (week 20 data shown; *P* = .004; see Figure 2(b)). Additionally, in the tail motor nerve, diabetic animals exposed to cold had a subtle but significant reduction in conduction velocity at 20 weeks (*P* = .017; see Figure 2(b)). 

In addition to tail nerve conduction, sciatic-tibial motor and sensory nerve conduction velocities were measured at one time point (16 weeks). Diabetic animals in both groups (room temperature environment and cold exposed) had significantly slower sciatic-tibial motor nerve conduction velocity than nondiabetic controls (*P* = .007; see Figure 2(c)), but no difference was seen between the cold exposed- and room temperature-diabetic groups. Similarly, nondiabetic room temperature and cold-exposed animals had normal sciatic-tibial sensory conduction velocities (53.3 +/− 4.0 m/s and 52.2 +/− 4.0 m/s); diabetic room temperature animals demonstrated mild slowing (48.9 +/− 4.6 m/s) of conduction velocity; diabetic cold-exposed animals demonstrated further slowing (46.5 +/− 4.6 m/s), however none of these differences were significant.

### 3.3. Animal Temperature

**Figure 3:**
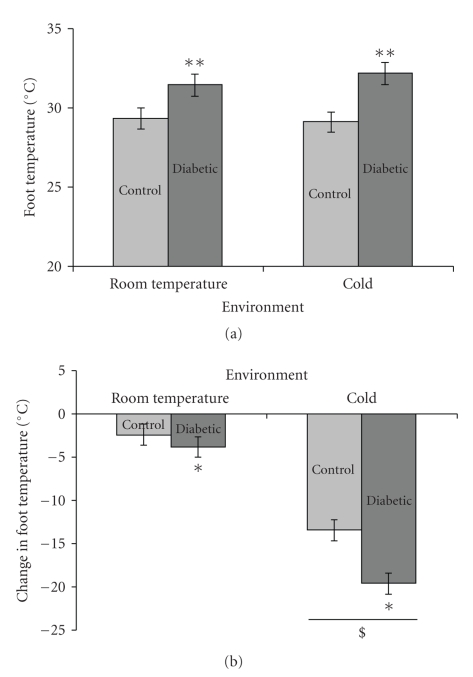
Foot temperature measurements. Graphs of foot temperature at room temperature and reduction in foot temperature during environmental exposure (*n* = 7 each control group, *n* = 6 each diabetic group). (a) Foot temperature was higher in diabetic rats, in both the room temperature and cold exposed groups, when measured at room temperature. (b) During environmental exposure, animals in the cold exposed groups (both nondiabetic controls and diabetics) had the greatest decrease in foot temperature. Diabetic animals (both room temperature and cold exposed) had a greater change in foot temperature compared to nondiabetic controls; there was also a trend for the diabetic animals exposed to a cold environment to have the greatest decrease in foot temperature (*P* = .089). Mean ± SEM. **P < .001 versus nondiabetic controls,  *P = .016 versus nondiabetic controls, ^*$*^ *P* < .001 versus room temperature.

There were no significant differences in core temperature (*P* = .35) or tail temperature (*P* = .52) among any groups after 20 weeks of treatment (data not shown). However, after 20 weeks of treatment, diabetic animals in both the room temperature and cold exposed environments had a significantly higher foot temperature than controls, when measured at room temperature (*P* < .001; see Figure 3(a)). Although diabetic animals had a higher foot temperature than nondiabetic controls, both diabetic and nondiabetic animals achieved the same foot temperature when exposed to a cold environment (*P* = .54). Thus, animals exposed to a cold environment (both diabetic and nondiabetic control groups) showed a greater reduction in foot temperature compared to animals in the room temperature group after exposure to their respective environments (week 20 data shown; *P* < .001; see Figure 3(b)). In addition, diabetic animals in both temperature groups had a larger change in temperature during the exposure periods than nondiabetic controls (week 20 data shown;  *P* = .016; see Figure 3(b)).

### 3.4. Pathology

*Sural Nerve.* There were no significant differences in axon density (*P* = .40; data not shown) or g quotients (*P* = .54; data not shown) among the four groups.

**Figure 4:**
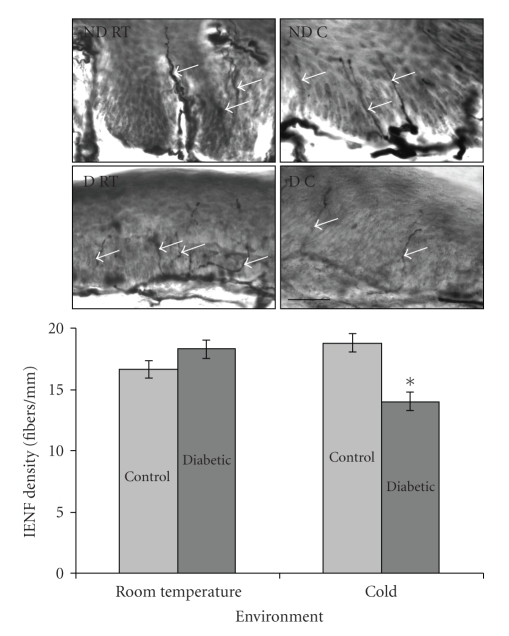
PGP 9.5 stained skin and skin fiber density. Photomicrographs showing PGP 9.5-stained sections. Pictures were taken at 40X, scale bar = 50 *μ*m. ND RT: nondiabetic room temperature, ND C: nondiabetic cold, D RT: diabetic room temperature, D C: diabetic cold. Graph of IENF densities shows that diabetic animals exposed to a cold environment had the lowest IENF densities (*n* = 7 each control group, *n* = 6 each diabetic group). The data suggest that diabetic animals exposed to a cold environment have the smallest number of stained nerve fibers in the skin. White arrows indicate PGP 9.5-positive fibers. Mean ± SEM. *P = .037 for the interaction term.

*IENF Density.* After 20 weeks of treatment, diabetic animals exposed to a cold environment had the lowest IENF density (*P* = .037; see Figure 4).

### 3.5. Nitrotyrosine ELISA

*Sural Nerve.* There were no significant differences in nitrotyrosine measurements among any group (*P* = .20; data not shown).

*Skin.* After 20 weeks of treatment, diabetic animals exposed to a cold environment had, on average, higher levels of nitrotyrosine in the skin (diabetic cold = 3.11 OD versus diabetic room temperature = 2.89, nondiabetic cold = 2.80, nondiabetic room temperature = 2.99; *P* = .047; data not shown).

## 4. Discussion

This study provides early evidence for a subtle detrimental effect of repeated cold exposure on the peripheral nerves of diabetic rats, possibly through reactive oxygen species (ROS) formation, as evidenced by elevated nitrotyrosine on skin ELISA. Although standard motor and sensory conduction studies in the tail and sciatic-tibial nerve revealed only small differences between cold- and room temperature-exposed animals, the H-reflex and IENF data are more suggestive. 

Each of the major pathways considered potentially causative of DPN may be enhanced by cold exposure. For example, cooling and excessive temperature fluctuations have been shown to enhance production of ROS in otherwise healthy nerves. Constant cooling in the 10–12^o^C range can increase ROS formation in normal rat nerves and reduce the activity of glutathione, which is important in the degradation of ROS [18]. In fact, increased levels of ROS and decreased levels of glutathione lead directly to decreased nerve conduction velocity [19, 20], supporting the idea that increased ROS levels may account for the increased H-reflex latency and the other electrophysiologic differences observed. Unfortunately, we were not able to demonstrate elevated levels of nitrotyrosine in the nerve to support this concept; however, it is possible that other markers of oxidative injury that were not measured were increased. In contrast, we did identify elevated levels of nitrotyrosine in the skin of diabetic cold animals, adding support to the concept that ROS may be involved in intraepidermal nerve fiber degeneration. This idea is also supported by other work in which diabetic animals lacking poly (ADP-ribose) polymerase, an enzyme believed to contribute to oxidative damage, did not show a decrease in skin nerve fibers compared to diabetic wild-type controls [21].

Animals exposed to intermittent cooling in the 1–5°C range experience rapid and severe block of neuronal conduction and edema in the myelin sheath of large neurons and endoneurial blood vessels [22, 23]. Such changes are typical of those seen in nerve tissue after ischemia-reperfusion injury [24] in which ROS formation plays a major role [25]. In fact, diabetic animals are more susceptible to nerve damage as a result of ischemic injury [10, 11], though typically the mechanism of ischemia in animal models is acute and severe (e.g., temporary ligation of major arteries in the hindlimb). In the current study, repeated cold exposure may have produced a mild form of chronic ischemia-reperfusion injury, accounting for the increase in ROS observed. It is of interest that other work, mainly in the realm of stroke and spinal cord injury [26, 27] has shown that hypothermia reduces ischemic-reperfusion injury. However, both the type of injury (acute and severe) and type of cooling applied (short-term) are quite different from that studied in these experiments in which cooling was continued for a period of many weeks in the setting of a gradually progressive disease process. 

Although our studies did not employ such low temperatures in the 1–5°C range, as described above, the results of this study suggest the possibility that ROS could play an important role at higher temperatures, especially in susceptible diabetic nerves. Indeed, with exposure to more mild cooling in the 20°C range, elevations in plasma viscosity [28], red cell rigidity and volume [29], and reductions in leukocyte mobility [30] are seen, leading to a reduction in the rate of blood flow through vessels. As a consequence of reduced blood flow, there is a slowing of oxygen delivery and a reduction in the elimination of toxic metabolites. Similarly, concentrations of endothelial-derived relaxing factors (e.g., nitric oxide, prostacyclin) and constricting factors (e.g., endothelin, angiotensin II), as well as blood vessel responses to these factors, are also temperature dependent [31–33], potentially leading to reduced blood flow. Reduced axonal transport also could play an important role. For example, partial failure of fast axoplasmic transport occurs at a temperature of 11°C, with slowing ensuing at considerably higher temperatures [6]. Reductions in the rate of slow and retrograde transport have also been demonstrated with even relatively mild cooling (in the 14°C to 30°C range) [7, 34, 35]. Finally, enhancement of protein kinase C activity can also occur with cooling [8, 36]. 

These studies were performed at a variety of temperatures, from as low as 1°C to as high as 30°C. Our study looked at the effect of cooling only at 10 ± 2°C, which is relatively cold for human exposure during routine activities. However, exposure to ambient temperatures equal to or lower than 10°C is possible in temperate areas during the winter months. Upon exposure to extremely low ambient temperatures (those below 0°C) skin surface temperatures as low as 12°C and 18°C have been measured, even during periods of activity [37, 38]. Therefore, while the cold temperature used in this study may seem extreme for human exposure, it is possible for skin to achieve a lower than normal temperature in certain geographic locales.

Given the fact that substantial cold exposure occurs in humans living in climates with cold winters and given the plethora of mechanisms by which cooling could enhance many of the DPN pathogenic pathways, it is perhaps not surprising that we have found evidence for at least a subtle effect. Moreover, thermoregulatory impairment in advancing DPN may also allow foot temperature to decrease further than normal and for more extended periods of time. Recent work that we have completed in human subjects supports that such abnormalities in thermoregulatory control are easily observed [39]. The data presented here (Figure 3(b)) support the possibility of this association in that there was a trend for the diabetic animals exposed to cold to develop a relatively greater reduction in foot temperature than the nondiabetic controls exposed to cold. 

Although we identified changes in H-reflex measurements, we did not identify clear pathology in sural nerve axon density at 20 weeks. This is not unexpected, in part, because H-reflex abnormalities have been reported as a very early indicator of DPN, even in asymptomatic patients [40] and similarly negative nerve pathology results have been found in STZ-induced diabetes [41, 42]. Additionally, we found a decrease in IENF density only in the diabetic cold group but unexpectedly not in the diabetic room temperature group. Although some investigators have found a reduction in IENF in the rat STZ model of DPN, direct comparisons to published data are difficult, due to differences in duration of diabetes and rat strain [43, 44]. Moreover, an earlier study assessing IENF density in the foot pads of STZ-induced diabetic rats actually showed an increase in density [45]. 

In this study, we assessed just a single cold temperature range (10 ± 2°C) and so additional studies assessing several temperatures, including perhaps warmer ones, may be warranted. Moreover, our choice of 23°C for the room temperature cohorts may not have been ideal since that temperature may already be considerably lower than most animals experience regularly, given the presence of bedding and the warmth/insulation this provides. Thus, future animal work will need to focus on the establishment of a dose-response effect with cooling, the potential effects of warming in ameliorating DPN, and, of course, more detailed studies of the mechanisms underlying this phenomenon.

## 5. Conclusion

The aim of this study was to evaluate the effects of repeated cold exposure on the development of DPN in the streptozotocin model of rat diabetes. The results of this study indicate that such exposure in diabetic animals exacerbates DPN, as measured by electrophysiological assessments and epidermal nerve fiber density. Additionally, cold exposure in diabetic animals leads to an increase in skin nitrotyrosine levels, implicating reactive oxygen species formation as one potential mechanism underlying this effect. These results suggest that limiting cold exposure of the distal extremities in human diabetic patients may help slow onset and progression of DPN.

## Figures and Tables

**Table 1:** Rat status at 20 weeks.

Group	Weight (g)	Glucose (mg/dL)	HbA_1c_ (%)
	mean ± SEM	mean ± SEM	mean ± SEM
Nondiabetic, room temperature (*n* = 7)	533 ± 14	125 ± 22	4.8 ± 0.3
Nondiabetic, cold-exposed (*n* = 7)	532 ± 14	125 ± 20	5.1 ± 0.3
Diabetic, room temperature (*n* = 6)	372 ± 16*	345 ± 22*	10.7 ± 0.3*
Diabetic, cold-exposed (*n* = 6)	379 ± 16*	352 ± 22*	10.9 ± 0.3*

*P < .0001 versus nondiabetic.
